# Alterations in tissue microRNA after heat stress in the conscious rat: potential biomarkers of organ-specific injury

**DOI:** 10.1186/s12864-019-5515-6

**Published:** 2019-02-15

**Authors:** Matthew G. Permenter, Bonna C. McDyre, Danielle L. Ippolito, Jonathan D. Stallings

**Affiliations:** 1 Excet, Inc., Fort Detrick, MD 21702-5010 USA; 2 Oak Ridge Institute for Science and Education, Fort Detrick, MD 21702-5010 USA; 3 U.S. Army Center for Environmental Health Research, Fort Detrick, Maryland, MD 21702-5010 USA

**Keywords:** Heat stress, Heat shock, miRNA, Transcriptomics, Biomarker

## Abstract

**Background:**

Heat illness remains a significant cause of morbidity in susceptible populations. Recent research elucidating the cellular mechanism of heat stress leading to heat illness may provide information to develop better therapeutic interventions, risk assessment strategies, and early biomarkers of organ damage. microRNA (miRNA) are promising candidates for therapeutic targets and biomarkers for a variety of clinical conditions since there is the potential for high specificity for individual tissues and unique cellular functions. The objective of this study was to identify differentially expressed microRNAs and their putative mRNA targets in the heart, liver, kidney, and lung in rats at three time points: during heat stress (i.e., when core temperature reached 41.8 °C), or following a 24 or 48 h recovery period.

**Results:**

Rats did not show histological evidence of tissue pathology until 48 h after heat stress, with 3 out of 6 rats showing cardiac inflammation and renal proteinosis at 48 h. The three rats with cardiac and renal pathology had 86, 7, 159, and 37 differentially expressed miRNA in the heart, liver, kidney, or lung, respectively compared to non-heat stressed control animals. During heat stress one differentially expressed miRNA was found in the liver and five in the lung, with no other modulated miRNA after 24 h or 48 h in animals with no evidence of organ injury. Pathway enrichment analysis revealed enrichment in functional pathways associated with heat stress, with the greatest effects observed in animals with histological evidence of cardiac and renal damage at 48 h. Inhibiting miR-21 in cultured cardiomyocytes increased the percent apoptotic cells five hours after heat stress from 70.9 ± 0.8 to 84.8 ± 2.2%.

**Conclusions:**

Global microRNA and transcriptomics analysis suggested that perturbed miRNA due to heat stress are involved in biological pathways related to organ injury, energy metabolism, the unfolded protein response, and cellular signaling. These miRNA may serve as biomarkers of organ injury and potential pharmacological targets for preventing heat illness or organ injury.

**Electronic supplementary material:**

The online version of this article (10.1186/s12864-019-5515-6) contains supplementary material, which is available to authorized users.

## Background

Heat illness is a continuum of disorders caused by hyperthermia, and includes clinical outcomes such as heat cramps, heat exhaustion, heat injury, and heat stroke [1–3]. Excessive physiological strain or heat load in combination with other strains (e.g., work, fatigue, excessive clothing and dehydration) contribute to the onset of heat illness [4, 5]. Heat illness is preventable by proper hydration and cooling, but heat illness remains a significant public health concern. Between 1997 and 2006, an estimated 54,983 exertional heat-related injuries were treated in emergency departments in the United States nationally [6]. Between 1999 and 2009, an average of 658 total heat-related deaths occurred in the United States annually [7]. Improved risk assessments and heat-related injury biomarkers are needed to evaluate heat-related injuries and prevent deaths.

At the cellular level, the in vitro heat shock and stress response is well known [8, 9]. Central to the heat shock response is the activation of the heat shock factor (HSF) transcription factor, leading to the production of heat shock proteins (HSPs), which chaperone misfolded proteins [10–12]. Heat stress inhibits DNA synthesis, transcription, RNA processing, and translation, potentially leading to cell cycle arrest. Excessive heat causes protein denaturation and aggregation with subsequent protein degradation by proteasomes and lysosomes. Additionally, heat stress interferes with energy metabolism, resulting in a decrease in ATP. Our previous modeling studies demonstrate that the heat shock response is initially acute and self-limiting at lower temperatures, due to the negative feedback loop between HSF and HSP [13]. As temperature and exposure times increase, transcriptional and translational efficiency decrease; the negative feedback loop loses efficacy; and HSF activation predominates, leading to a much more persistent activation of the transcription factor.

The in vivo heat stress response at the cellular and systemic levels is not well characterized as the physiological response to hyperthermia [9, 14]. In our recent panomics studies [15, 16], we reported that rats with heat-related cardiac pathology had enriched pathways associated with proteotoxic stress and/or large functional complexes. Cardiac injury coincided with significantly enriched Kyoto Encyclopedia of Genes and Genomes (KEGG) pathways [17] associated with oxidative phosphorylation, antigen processing and presentation, and cardiac muscle contractility. The enrichment is consistent with protein folding disorders, mitochondrial dysfunction, and perturbation in cellular energetics. These results are concordant with studies published by Ciryam *et .al* [18], citing proteotoxic stress and protein aggregation as a mechanism for degenerative diseases caused by the unfolded protein response in humans.

In the present study, we investigate microRNA (miRNA) regulators of the transcriptomic and proteomic response to heat stress reported in our previous study. miRNA are approximately 22 nucleotide sequences which epigenetically bind to and predominately negatively regulate mRNA transcription. miRNA may be tissue-specific, making them attractive therapeutic targets for a variety of diseases [19]. We hypothesized that distinct patterns of miRNA expression correspond to heat stress, recovery, and the cardiac and renal pathology observed in our conscious rat model of heat stress. Further, we predicted that mapping these differentially expressed miRNA and their putative mRNA targets to cellular pathways would elucidate potentially novel cellular mechanisms and pharmacological targets for heat illness.

## Methods

### Animal model and tissue collection

In vivo rat experiments were performed at the United States Army Research Institute of Environmental Medicine. Husbandry, exposure to heat, tissue collection, physiological and hematological parameter collection, and histopathologic analysis were conducted as previously described in Rakesh et al. (2013) and Stallings et al. (2014). Briefly, Fischer 344 rats (Charles River Laboratories, Stone Ridge, NY) were heated until their core temperature reached 41.8 °C as measured by implanted telemetry probe (T_c,max_). The animals were euthanized and heart, liver, kidney, and lung tissue were collected at T_c,max_ and 24 and 48 h after recovery (*n* = 6 per cohort). Control animals were placed in an incubator maintained at room temperature, and rats were sacrificed at concurrent time points (n = 6 total). Portions of each tissue were both flash frozen for cryopreservation at − 80 °C in cryovials and also prepared for histology by fixation in 10% formalin and hematoxylin and eosin staining. Tissue pathology was assessed by a board-certified veterinary pathologist.

### Isolation of RNA

Frozen lung, liver, and kidney tissue were placed on dry ice, cut into aliquots with a sterile scalpel on a pre-chilled titanium block, and placed in new pre-chilled tubes. The block was washed with RNase Zap (Ambion, Life Technologies, Grand Island, NY) between samples. While working on dry ice, whole tissues (~ 25 mg) from the lung, liver, heart, and kidney were cut from each sample and placed in 700 mL of QIAzol Lysis Reagent. Samples were homogenized with a TissueLyzer LT (Qiagen, Valencia, CA) for 5 min at 25 Hz twice, allowed to sit at room temperature for 5 min, and then placed at − 80 °C.

We homogenized heart tissue with a Spex 6750 Freezer Mill with three sample microvials (Spex SamplePrep, Metuchen, NJ). Approximately 1 cm^3^ of heart tissue was excised, dipped into liquid nitrogen, and then placed into pre-assembled and pre-chilled microvials containing 200 μL of QIAzol. An impactor was added to each microvial, securely capped, and placed in the mill. The Freezer Mill settings were as follows: T1 run time = 3 min, T2 intermission = 1 min, T3 pre-cool = 5 min, rate = 15, and cycles = 2. After completion, we added 500 μL of QIAzol to each vial, allowed the vial to sit at room temperature for 5 min, transferred the sample to a microfuge tube, and then placed it at − 80 °C.

The following day, we allowed all samples to thaw and isolated RNA using the miRNeasy 96 kit (Qiagen) according to the manufacturer’s instructions. The quality and quantity of RNA samples were evaluated with a 2100 Bioanalyzer (Agilent Technologies, Santa Clara, CA) using the Agilent RNA 6000 Nano Reagents and a multi-well Nanodrop 8000 spectrophotometer (Thermo Fisher Scientific, Waltham, MA).

### Affymetrix gene Array

We prepared cDNA from total RNA using the Ambion Whole Transcript (WT) Expression Kit (Ambion) as previously described [15]. The cDNA was fragmented, labeled, hybridized, stained, and washed per the manufacturer’s recommendations (Affymetrix, Santa Clara, CA). Samples were then applied to the Rat Gene 1.1 ST 16 Array Plate or 24 Array Plate and placed in the GeneTitan System (Affymetrix) according the manufacturer’s recommendations. Of the 144 arrays, 2 liver arrays, 1 lung array, and 1 heart array did not pass quality control checks and were excluded from further analysis.

### Small RNA library construction

Small RNA libraries were constructed using the TruSeq small RNA library preparation kit (Illumina, San Diego, CA) according to the manufacturer’s instructions. Total RNA was ligated to 3′ and 5′ adaptors containing bases targeting small RNAs and sequencing primer sequences, respectively. Adaptor-ligated small RNA molecule enrichment was achieved by reverse transcription followed by limited-cycle polymerase chain reaction with sample-specific barcode tags incorporated at this step. The quality and concentration of newly constructed libraries were assessed with the Agilent 2100 Bioanalyzer. Small RNA libraries constructed from the same type of tissue were pooled together in equimolar amounts and separated on 6% Novex Tris-Borate-EDTA gels (Life Technologies, Grand Island, NY) for size selection. Gel-purified sequencing libraries were quantified using the KAPA Library Quantification Kit (Kapa Biosystems, Woburn, MA) and diluted to a final concentration of 2 nM.

### Next-generation sequencing

Cluster generation of the small RNA sequencing libraries and a PhiX control library was performed using an automated cBot system (Illumina). All sequencing libraries were loaded onto a TruSeq single-read flow cell v3 at a concentration of 12 pM. The small RNA sequencing libraries were spiked with 1% (vol/vol) PhiX control library and loaded onto four individual flow cell lanes based on the tissue of origin as each lane contained multiplexed sequencing libraries. The small RNA libraries were sequenced using the Illumina HiScan SQ system for a total of 58 cycles. The performance of the sequencing run and quality of raw sequence data were assessed based on the reports created by the Illumina Sequence Analysis Viewer. All reported parameters met the quality assessment criteria recommended by the manufacturer.

### Sequencing data processing

To obtain the spectrum of miRNA in samples, the sequence files (Fastq files) were preprocessed before being mapped to the miRNA database. In the preprocess step, adapter sequences were trimmed with Cutadapt software (version 2.6) [20]. In addition, low complexity sequences, such as homopolymer sequences, were removed with the Prinseq tool (version 0.20.4) [21]. To reduce the time required for sequence mapping, the redundant sequences in the file were collapsed with the FASTX collapse script obtained from the FASTX toolkit (version 0.0.13) [22]. The number of reads for each unique sequence were calculated and used in summarizing the mapping read count. The unique read sequences were then mapped against the reference rat miRNA sequence database from miRBase (Release 19) under the perfect match condition [23–27]. The sequencing data can be found in the Gene Expression Omnibus with the accession number GSE81331.

### Data and pathway analysis

A total of 1040 miRNA was identified. Differentially expressed miRNA were determined using the R package edgeR [28, 29] with each tissue analyzed individually. To remove miRNA that were not expressed above background, we removed any miRNA that did not have at least 5 reads in all samples of at least 1 condition. Differentially expressed miRNA were chosen as those with an FDR < 0.05 when tested against a 1.25 fold change cutoff using the glmTREAT function of edgeR.

KEGG pathway analysis was conducted in DAVID [30, 31] using human homologs of miRNA and mRNAs. We used differentially expressed miRNA for each condition and any mRNA in the same condition from our previous work [15] that changed by at least 1.5-fold for this analysis. Using the miRNA target analysis in Ingenuity Pathway Analysis (IPA) software (Qiagen, Valencia, CA) [32], we identified mRNA from the microarray experiment that were predicted or validated targets of the differentially expressed miRNA. We limited the targets to those that were changing in the opposite direction of the miRNA. KEGG pathway analysis was then conducted on the union of all targeted mRNA by all differentially expressed miRNA in a condition using DAVID. A *p*-value less than 0.05 was considered statistically significant.

### miR-21 transfection and apoptosis assay

We also conducted in vitro experiments to further investigate the role that miR-21 plays in the response to heat stress as it was one of the most dysregulated miRNA. The rat myoblast-derived cell line H9c2 (American Type Culture Collection, Manassas, VA) was grown in Dulbecco’s Modified Eagle’s Medium (Lonza, Walkersville, MD) containing 10% fetal bovine serum (Invitrogen, Carlsbad, CA) and 10 mL Glutamax (Invitrogen) in T75 flasks incubated at 37 °C with 5% carbon dioxide. Cells were plated in six-well plates at a density of 30,000 cells/well and after 4 days were transfected with either a miRNA mimic to increase the expression of miR-21, an inhibitor to decrease the expression, or the transfection vehicle only as a control. Transfection was performed using a mirVANA miRNA mimic and inhibitor (mmu-miR-21a-3p, Life Technologies) and Lipofectamine RNAiMAX reagent per the manufacturer’s instructions. Successful transfection was verified by quantitative PCR (data not shown). One day after transfection, cells were either heated to 47 °C or remained at 37 °C, and apoptosis was measured after 2, 3, 4 and 5 h using the Guava Nexin Assay on the easyCyte HT system (Guava, Hayward, CA) per the manufacturer’s instructions. A two sample t-test determined statistical significance (*p* < 0.05).

## Results

### Physiological profiles and histology

Physiological profiles of the rats used in this study were consistent with dehydration and/or kidney function impairment (including increased blood urea nitrogen and change in electrolytes at T_c,Max_) as detailed in our previous reports [15, 16, 33]. Rats in the heat-stressed cohorts reached 41.9 ± 0.1 °C (T_c,Max_) in 2–3 h [33]. As previously reported, no heat-related pathologies were observable by histology at T_c,max_ or 24 h after heating (Additional File 1 and [15, 16]). At 48 h after heating, three of the six heat-stressed rats had histological evidence of moderate cardiac inflammation and cardiomyocyte degeneration with increased kidney proteinosis (Additional File 1). The three animals with evidence of organ injury demonstrated transcriptomic profiles different from the other heat stressed rats at 48 h and controls in our previous work. Therefore, in order to identify biomarkers and mechanisms leading to organ injury, these three animals were analyzed as a separate treatment group.

### Identification of differentially expressed miRNA

**Fig. 1:**
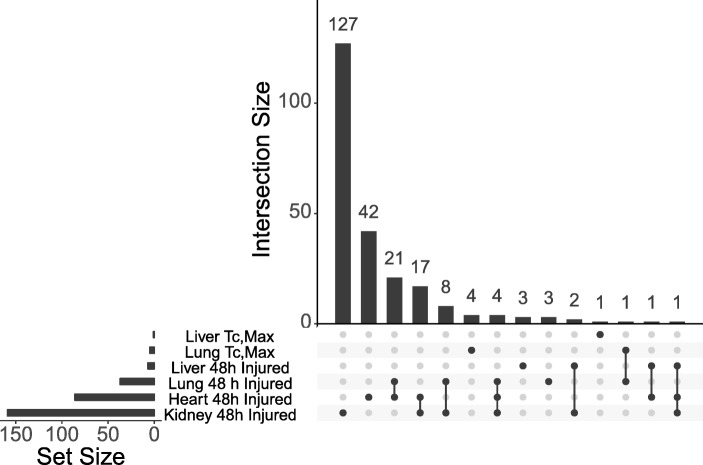
Differentially expressed miRNA are mostly time and tissue specific. The bar chart represents the number of differentially expressed miRNA in each of the data sets indicated by a filled dot below. If the miRNA is modulated in more than one time point or tissue, multiple dots are darkened and connected by a line. The set size indicates the total number of differentially expressed miRNA at each tissue/time point

**Fig. 2:**
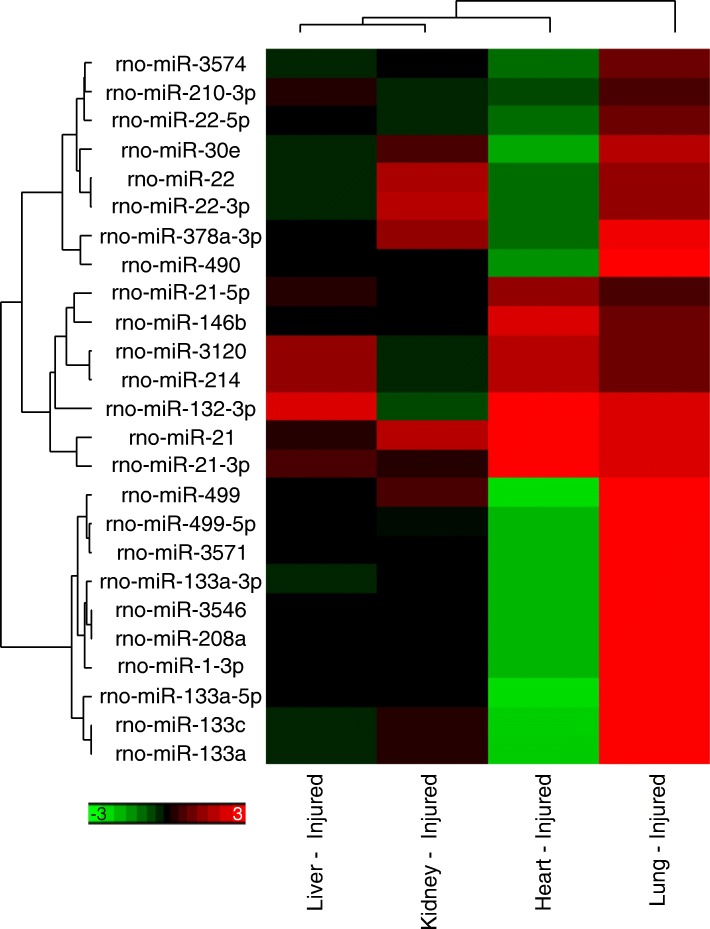
miRNA differentially expressed in multiple tissues. Three of the miRNA differentially expressed in the lung, heart, and kidney of the injured animals (rno-miR-22, rno-miR-22-3p, and rno-miR-378a-3p) were down-regulated in the heart, but upregulated in the kidney and lung. This is also seen with the miRNA differentially expressed in both the heart and lung. Of the 25 shared miRNA, 15 were regulated in the opposite direction, with all of them down-regulated in the heart, and up-regulated in the lung. The color of the hierarchical clustering shows the log2 fold change

We identified miRNA isolated from the heart, kidney, liver, and lung of heat-stressed rats by RNA sequencing. Among all samples, we identified 1040 *Rattus norvegicus* miRNA, of which 292, 397, 256, or 426 miRNA in the heart, kidney, liver, or lung, respectively, had more than 5 reads in all samples of at least one condition. We removed the three animals with evidence of cardiac and kidney proteinosis at 48 h from the analysis and identified differentially expressed miRNA at T_c,Max_, 24, and 48 h with an FDR < 0.05 tested against a fold-change threshold of 1.25 (Additional File 2). To identify potential biomarkers specific to organ injury, we identified differentially expressed miRNA with an FDR < 0.05 tested against a fold-change threshold of 1.25 in the three animals with cardiac and renal lesions after 48 h (Table 1 and Additional File 2). Overall, few miRNA were perturbed in tissues of animals without histological evidence of injury at all time points. However, low sample number, especially in the stratified 48 h groups, results in a lower powered study, limiting the ability to detect differentially expressed miRNAs. One miRNA in the liver and five miRNA in the lung were differentially expressed at T_c,max_, while no miRNA were changing in any other time point or tissue of the uninjured animals. However, in the animals with evidence of injury 86, 7, 159, and 37 miRNA were differentially expressed in the heart, liver, kidney, or lung, respectively. The majority of the differentially expressed miRNA were tissue specific (Fig. 1). The greatest overlap, with 21 shared modulated miRNA, was between the lung and heart of the injured animals. Only five total miRNA were shared by more than two conditions with four shared between the lung, heart, and kidney of the injured animals and one shared by the liver, heart, and kidney of the injured animals. Interestingly, three of the miRNA shared between the lung, heart, and kidney of the injured animals (rno-miR-22, rno-miR-22-3p, and rno-miR-378a-3p) were down-regulated in the heart, but upregulated in the kidney and lung. The pattern continues with the miRNA differentially expressed in both the heart and lung of the injured animals. Of the 25 shared miRNA, 18 were regulated in the opposite direction, with all of them down-regulated in the heart, and up-regulated in the lung in the injured animals (Fig. 2).

**Table 1:** Number of differentially expressed miRNA per condition

Tissue	Timepoint	miRNA
Heart	T_c,max_	0
	24	0
	48 - uninjured	0
	48 - injured	86
Liver	T_c,max_	1
	24	0
	48 - uninjured	0
	48 - injured	7
Kidney	T_c,max_	0
	24	0
	48 - uninjured	0
	48 - injured	159
Lung	T_c,max_	5
	24	0
	48 - uninjured	0
	48 - injured	37

Differentially expressed miRNA were identified in the liver, kidney, lung, and heart of heat stressed animals at 3 time points. A cohort of animals with histopathological evidence of injury at 48 h were analyzed separately from those with no evidence of injury

### Pathway analysis

To further understand the biological implications of the differentially expressed miRNA, we conducted an unsupervised pathway enrichment. We first identified the mRNA targets that were changing by at least 1.5 fold in the opposite direction of the perturbed miRNA (Additional File 3) from our previous work [15]. The pathway analysis was then conducted using the union of all mRNA targets of one condition. We identified 93, 1, 19, 3 and 8 significantly enriched KEGG pathways in the heart of the injured animals, liver at T_c,Max_, kidney of the injured animals, lung at T_c,Max_, and the lung of the injured animals, respectively (Table 2 and Additional File 4). No enriched pathways were common to all conditions. One enriched pathway, the p53 signaling pathway, was enriched in the all tissues of the injured animals suggesting a shared response to heat injury.

**Table 2:** Selected enriched KEGG pathways

Condition	Pathway	# of genes	p-value	Benjamini corrected p-value
Heart 48 h - Injured	Biosynthesis of antibiotics	62	< 0.001	< 0.001
	Metabolic pathways	237	< 0.001	< 0.001
	Insulin resistance	36	< 0.001	< 0.001
	cGMP-PKG signaling pathway	48	< 0.001	< 0.001
	Fatty acid metabolism	23	< 0.001	< 0.001
	PI3K-Akt signaling pathway	78	< 0.001	< 0.001
	Leukocyte transendothelial migration	37	< 0.001	< 0.001
	Carbon metabolism	37	< 0.001	< 0.001
	Citrate cycle (TCA cycle)	16	< 0.001	< 0.001
	Focal adhesion	53	< 0.001	< 0.001
	Arrhythmogenic right ventricular cardiomyopathy (ARVC)	23	< 0.001	0.001
	PPAR signaling pathway	23	< 0.001	0.004
	Protein digestion and absorption	25	< 0.001	0.005
	Hypertrophic cardiomyopathy (HCM)	23	0.001	0.006
	Dilated cardiomyopathy	22	0.003	0.021
Liver Tc,max	Endocytosis	4	0.016	0.537
Kidney 48 h - Injured	Complement and coagulation cascades	14	< 0.001	< 0.001
	Cell cycle	16	< 0.001	< 0.001
	p53 signaling pathway	9	< 0.001	0.003
	Bile secretion	9	< 0.001	0.003
	PPAR signaling pathway	9	< 0.001	0.004
	FoxO signaling pathway	10	0.001	0.037
	Progesterone-mediated oocyte maturation	8	0.001	0.043
	MicroRNAs in cancer	9	0.006	0.141
	Small cell lung cancer	7	0.006	0.133
	Carbohydrate digestion and absorption	5	0.007	0.145
	Biosynthesis of unsaturated fatty acids	4	0.017	0.265
Lung Tc,max	TNF signaling pathway	4	0.026	0.974
	Pathways in cancer	7	0.029	0.863
	NOD-like receptor signaling pathway	3	0.043	0.866
Lung 48 h - Injured	Calcium signaling pathway	8	0.001	0.145
	PPAR signaling pathway	5	0.005	0.266
	Oocyte meiosis	5	0.016	0.499
	p53 signaling pathway	4	0.024	0.548
	Signaling pathways regulating pluripotency of stem cells	5	0.035	0.610
	Protein digestion and absorption	4	0.044	0.627
	Neuroactive ligand-receptor interaction	7	0.044	0.571
	Tyrosine metabolism	3	0.047	0.542

The top ten and selected enriched KEGG pathways for each condition are shown with *p*-values and adjusted p-values. The number of genes listed are unique genes that are targets of the differentially expressed miRNA in that condition and contribute to the enrichment of the pathway. The complete list of enriched pathways are listed in Additional File 4

### Modulation of miR-21 in cardiomyocytes

**Fig. 3:**
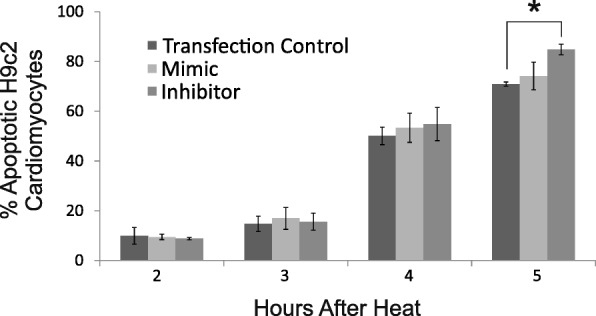
Effect of miR-21 expression on apoptosis. The y-axis shows the percentage of cells undergoing apoptosis. No protective effect of miR-21 against heat induced apoptosis was observed at any time point in H9c2 cells which over expressed miR-21. However, a significant increase in apoptosis in cells with decreased miR-21 expression compared to the transfection control was observed at 5 h, further suggesting that miR-21 plays a critical role in the heat stress response

miR-21 was identified as one of the most differentially expressed miRNA in the cardiac-injured animals and was also modulated in the lung and kidney of the injured animals. To further explore the role that miR-21 plays in the heat stress response, we manipulated the levels of miR-21 in an in vitro cell model. We hypothesized that increasing the expression level of miR-21 plays a protective role against heat stress, whereas decreasing the expression is detrimental. Therefore, we transfected H9c2 cells with either a miR-21 mimic or a miR-21 inhibitor and measured apoptosis induced by heat stress. Modulated expression of miR-21 significantly affected apoptosis in response to heat stress at 5 h after heating, in which apoptosis increased from 70.9 ± 0.8% to 84.8 ± 2.2% (*p* < 0.05) (Fig. 3).

## Discussion

Heat stress is known to affect cellular function through protein misfolding and aggregation, the degradation of these damaged proteins, and the disruption of metabolism leading to a decrease in ATP, and the activation of cell signaling pathways [9, 34]. Many of the enriched KEGG pathways and differentially expressed miRNA are involved in these well-studied responses to heat stress. These processes are especially evident in the animals with heat induced organ injury. This study uses a global miRNA/mRNA analysis approach to place miRNA and their presumptive targets within the context of biological pathways. Our study offers a systems-based multi-organ approach to understanding miRNA involvement in the biomolecular basis of the heat stress response.

### Organ injury

Little to no evidence exists for organ injury in any early time point in any tissue. No major histopathological findings indicated injury in any tissue before 48 h. Additionally, no KEGG pathways or miRNA related to organ injury were enriched before 48 h in any tissue. However, in animals with histopathological evidence of injury at 48 h, miRNA that may be linked to organ injury were modulated in the kidney.

In the hearts of animals with injury, pathways associated with cardiomyopathy and organ injury were enriched. The KEGG pathways arrhythmogenic right ventricular cardiomyopathy, hypertrophic cardiomyopathy, and dilated cardiomyopathy are enriched in the heart tissue of the injured animals. These terms are not enriched at any time point in those animals without evidence of injury. Additionally, miR-208 and miR-499 have been reported to be indicators of cardiac injury or modulated in heart disease [35, 36]. The pathways related to cardiomyopathy enriched solely in the heart tissue of animals with histopathological evidence of injury further suggest that these three animals sustained a more severe injury and/or failed to recover compared with the other, uninjured animals at 48 h. The up-regulation of particular miRNA with a failure to recover would be particularly useful for identifying individuals who are at greater future risk of heat illness or organ injury following an earlier episode of heat stress.

Furthermore, some of the differentially expressed miRNA have been implicated in heart disease or have been identified as potential biomarkers. In mice, the miR-29 family regulates profibrotic genes and is also modulated after myocardial infarction [37]. In our work rno-miR-29c, rno-miR29c-3p, and rno-miR-29b-3p are all down-regulated and would therefore enhance the fibrotic response, indicating a progression toward heart failure [36]. miR-208 and miR-499 play important roles in the heart and are at most minimally detectable in the plasma of healthy humans. However, both of these miRNA are elevated in the plasma of both an animal model and human patients with acute myocardial infarction, with miR-208 being the more reliable biomarker for injury [36]. In our data, rno-miR-499, rno-miR-499-5p, rno-miR-208a, and rno-miR-208a-5p are down-regulated in the heart of the injured animals. Interestingly, rno-miR-208a, rno-miR-499, and rno-miR-499-5p are all up-regulated in the lung of the injured animals. Additionally, miR-92a, which is thought to play a role in angiogenesis, is up regulated after the induction of acute myocardial infarction in a mouse model, which is reflected by the up-regulation of rno-miR-92a-3p in the heart of the injured animals [38]. These preclinical studies suggest that there is a set of miRNA that may be useful for assessing cardiac damage from thermal injury as well as from other causes.

The differentially expressed miRNA support the histopathologic analysis indicating that kidney injury occurred in heat injured animals. miR-21, miR-20a, miR-199a-3p, and miR-194 have all been shown to be modulated in a mouse model of acute kidney injury [39]. In the study conducted by Godwin and colleagues, miR-21 and miR-20a were rapidly up-regulated, miR-199a-3p was up-regulated 3 days post-stress and continued to increase for the duration of the time course of 30 days, whereas miR-194 was rapidly down-regulated [39]. In our data, both rno-miR-21 and rno-miR-20a are also upregulated in the kidney of the injured animals. Rno-miR-21 is also up-regulated in the heart and lung of the injured animals, while rno-miR-20a is also up-regulated in the heart of the injured animals. As in the Godwin study, rno-miR-194-3p and rno-miR-194-5p are down-regulated in the kidney of the injured animals. Interestingly, rno-miR-199a-3p and rno-miR-199a-5p are down-regulated in the kidney of the injured animals, matching Godwin’s results, yet they are up-regulated in the liver of the injured animals. A review conducted in 2016 identified miRNA which were modulated in acute kidney injury among published studies [40]. Twenty-five of the differentially expressed miRNA in the kidney of the animals with injury were also identified in this review as potential biomarkers of acute kidney injury (Table 3). Even though there is a conflict in the direction of change in some of the miRNA compared with other studies, they may align more closely at later or different time points. Nevertheless, these differentially expressed miRNA may serve as indicators of organ injury.

**Table 3:** Markers of kidney injury

miRNA	Fold Change in Injured Kidney	Previously Reported Expression	Previous Sample Type
let-7a-1-3p	−1.8	Up in urine, down in tissue	Tissue, Urine
let-7b	1.5	Down	Blood
let-7e	2.4	No consensus	Tissue, in vitro
miR-10a	0.9	Up, down	Tissue, Urine, Blood
miR-125a-5p	1.1	Down	Tissue, in vitro
miR-142-3p	1.7	Up	Tissue, in vitro
miR-142-5p	−3.3	Up	Tissue, in vitro
miR-155	2.0	No consensus	Blood, Urine, Tissue, in vitro
miR-15b-5p	−1.8	Down	Tissue, in vitro
miR-182	−2.6	Up	Tissue
miR-183-5p	−2.0	Up in urine, down in tissue	Tissue, Urine
miR-191a-5p	−2.9	Up in urine, down in tissue	Tissue, Urine
miR-199a-3p	−2.0	Up	Tissue, in vitro
miR-200a	1.1	No consensus	Tissue, Blood, Urine
miR-21	1.6	No consensus	Blood, Urine Tissue, in vitro
miR-25-3p	−2.4	Up in urine, down in tissue	Tissue, Urine
miR-26a	−2.2	Down	In vitro, Tissue
miR-26b	−2.7	Down in tissue and blood, up in urine	Tissue, in vitro, Urine, Blood
miR-27a-3p	−2.9	Down	Blood
miR-30c-1	−3.5	Up	Tissue
miR-320	1.9	No consensus	Blood, Tissue, Urine
miR-328a-3p	1.3	Up in urine,down in tissue	Tissue, Urine
miR-335	−3.1	Up in urine,down in tissue	Tissue, Urine
miR-378a-5p	1.5	Up in urine,down in tissue	Tissue, Urine
miR-93-5p	−2.5	Up in urine, down in tissue	Tissue, Urine

Twenty-five of the differentially expressed miRNA in the kidney of the animals with injury have been previously identified in other studies as markers of kidney injury

The differentially expressed miRNA in the liver also reflect the histopathologic evidence of liver damage. The over expression of miR-199 has been associated with the progression of liver fibrosis in a previous study examining liver fibrosis in human liver biopsies and is up-regulated in our work in the liver of the injured animals [41]. Additionally, miR-146b was differentially expressed among the different grades of liver fibrosis and is up-regulated in this work in the liver of the injured animals [41]. Therefore, we confirm the differential expression of these two markers of organ injury confirmed by our histopathological findings.

### Energy metabolism

Heat stress is also known to alter energy metabolism, resulting in a net decrease in ATP production [8]. Accordingly, many of the differentially expressed miRNA and enriched KEGG pathways in our work suggest a disruption in energy production in the injured animals. While no pathways are enriched in the liver, metabolism related pathways are enriched in the kidney and heart of the injured animals.

Perhaps the greatest effect of the disruption of energy metabolism was seen in the heart of the injured animals. Many pathways related to energy metabolism are enriched in the heart of the injured animals including metabolic pathways, fatty-acid metabolism, and the citrate cycle. As seen in the liver, Acsl1 and as well as many other transcripts involved in fatty acid β-oxidation such as acyl-CoA dehydrogenase medium chain (*Acadm*), enoyl-CoA hydratase, short chain 1 (*Echs1*) and hydroxyacyl-CoA dehydrogenase (*Hadh*) are down-regulated. *Acadm* is predicted to be targeted by rno-miR-132-3p and rno-miR-222, *Echs2* by rno-miR-18a-5p and rno-miR-214, and *Hadh* by rno-31a-dp and rno-miR-19b-3p. With many transcripts involved in fatty acid β-oxidation being down-regulated with the levels of their predicted miRNA regulators increased, we see evidence of a sustained energy crisis in the heart of the injured animals and a potential decrease in ATP production.

In our microarray data, glucose transporter type 4 (*Slc2a4*) is down-regulated 9.5-fold in the heart of injured animals compared with control animals suggesting it may play a role in decreased metabolic activity due to heat stress [15]. SLC2A4 is primarily responsible for the intake of glucose into fat and muscle from peripheral tissues [42]. SLC2A4 is normally sequestered in internal compartments but is translocated to the plasma membrane upon stimulation by insulin through the phosphatidylinositol 3-kinase (PI3K)-Akt signaling pathway, which is enriched in the heart of the injured animals [43]. Additionally, rno-miR-20a-5p and rno-miR-31a-5p, which are predicted to target Slc2a4, are up-regulated in the heart of injured animals. This suggests that heat stress caused a prolonged decrease in energy metabolism involving a decrease in *Slc2a4* expression that may be influenced by an up-regulation of its predicted regulators, rno-miR-20a-5p and rno-miR-31a-5p.

The disruption of energy metabolism is also evident in the kidney of the injured animals as the carbohydrate digestion and absorption and biosynthesis of unsaturated fatty acids pathways are enriched. The four genes responsible for the enrichment of the biosynthesis of unsaturated fatty acids pathway are bile acid-CoA:amino acid N-acyltransferase (*Baat*), acyl-CoA thioesterase 7 (*Acot7*), 3-hydroxyacyl-CoA dehydratase 3 (*Hacd3*), and stearoyl-CoA desaturase (*Scd*). *Baat* is predicted to be targeted by rno-miR-143 and is down-regulated in the transcriptomic data. Rno-miR-143 and rno-miR-322-5p are predicted to target *Acot7* which is up-regulated. *Hacd3* is down-regulated and is predicted to be targeted by rno-miR-3588 and rno-miR-3585-5p. *Scd*, which is down-regulated, is predicted to be a target of many of the differentially expressed miRNA in the kidney of the injured animals including rno-let-7e, rno-miR-3588, rno-miR-351-5p, rno-miR-138-5p, rno-miR-200b-3p, rno-miR-22-3p, rno-miR-221-3p, rno-miR-27b-5p, rno-miR-324-5p, and rno-miR-410-5p. Overall, the dysregulation of the fatty acid biosynthesis and other energy metabolism related miRNA and transcripts suggests that the energy crisis and net decrease in ATP production continued in the kidney of the injured animals.

While it appears energy metabolism plays an immediate role in the response to heat stress, the greatest effect seemed to be in injured animals. This may suggest that the uninjured animals were able to recover fully from the stress, whereas the energy dysregulation continued in the animals with histopathological evidence of injury.

### Cell signaling

Various cell signaling pathways may also be perturbed as the animals either adapted to or were injured by heat stress. Many pathways related to cell signaling were enriched in multiple tissues and time points, although these pathways were especially evident in the injured animals.

Signaling pathways influenced by miRNA expression were effected at early time points in the lung. Both the TNF signaling and NOD-like receptor signaling pathways are enriched in the lung at T_c,max_. NOD-like receptors have been shown to be sensors of intracellular stress and infection [44]. The three transcripts that are differentially expressed in this pathway, heat shock protein 90 beta family member 1 (*Hsp90b1*), PYD and CARD domain containing (*Pycard*), and C-C motif chemokine ligand 5 (*Ccl5*), are predicted to be targeted by rno-miR-15b (*HSP90B1*) and rno-miR-122-5p (*Pycard* and *Ccl5*) suggesting these miRNA are involved in the regulation of the NOD-like proteins response to heat stress. A study by Imao and colleagues suggested that depending on the length of the heat stress event, TNF-α could promote or prevent liver cell death [45]. The modulated genes in the TNF signaling pathway are predicted to be targets of rno-miR-15b (prostaglandin-endoperoxide synthase 2, Jun proto-oncogene, and HNHc-like endonuclease) and rno-miR-92a-1-5p (*Ccl5*), suggesting these two miRNA are involved the regulation of the TNF signaling pathway perhaps in a return to homeostasis.

Peroxisome proliferator-activated receptors (PPARs) are involved in regulating multiple biological functions including energy metabolism. PPARs are transcription factors involved in the regulation of lipid and glucose homeostasis [46] and may also influence the transcriptional response to heat stress [47]. The PPAR signaling pathway is enriched in the heart, kidney, and lung of the injured animals. Differentially expressed miRNA in the heart of injured animals are predicted to target peroxisome proliferator-activated receptor gamma coactivator 1-alpha (*Ppargc1a*) while a miRNA modulated in the lung is predicted to target *Ppar-α*. *Ppargc1a* contains a predicted binding site for rno-miR-193-3p, whereas *Ppar-α* contains binding sites for rno-miR-22-3p. Both miRNA are up-regulated in their respective tissues and their targets are down-regulated in the transcriptomic data, suggesting an expected down regulation of PPARs. While PPARs are involved in lipid and glucose homeostasis, they also affect other pathways. PPAR-α also responds to chemical-induced inflammation, oxidative stress, and tissue growth [48]. Therefore, these miRNA may be involved in regulating energy metabolism or secondary effects in the heart of injured animals through the control of PPARs.

### Unfolded protein response

Heat stress can also cause an increase of denatured and aggregated proteins, which are then degraded through proteasomal and lysosomal pathways [34]. In the animals with histopathological evidence of injury, we observed evidence of a response to unfolded proteins, especially in the heart. In the heart and lung of the injured animals the protein digestion and absorption pathway is enriched, while the PI3K-Akt signaling pathway is enriched in the heart. Additionally, many modulated genes and the miRNA which target them are involved in the unfolded protein response. Many of the members of DNAJ heat shock protein family (*Hsp40*) are targeted by rno-miR-29c, rno-miR-34c-5p, rno-miR-214, rno-miR-351-5p, rno-miR-429, rno-let-7i-5p, rno-miR-193-3p, rno-miR-20a-5p, rno-miR-92a-3p, rno-miR-146b-5p, rno-miR-133a-5p, rno-miR-182, rno-miR-19b-3p, rno-miR-141-3p, rno-miR-9a-5p, or rno-miR-490. The majority of the miRNA regulation is resulting in a down-regulation of *Hsp40*, with the exception of *Dnajc2*, *Dnac3*, and *Dnajc9*. The DNAJ protein family is co- chaperone with heat shock protein 70, which are both then involved in protein folding and degradation [49]. Heat shock protein family A (*Hsp70*) member 9 (*Hspa9*) is also down-regulated in the transcriptomic data and is predicted to be a target of rno-miR-429. Additionally, members of the heat shock protein 90 family are modulated in our transcriptomic data [15] and are predicted to be targets of the differentially expressed miRNA. These transcripts are up-regulated and targets of rno-miR-9a-5p, rno-miR-1, and rno-miR-181d. While HSP40 and HSP70 seem to be involved in the early stages of protein folding, the HSP90 family of proteins are more specialized and involved in the late stages of folding, preventing the aggregation of proteins [50]. With most of the transcripts of *Hsp40* and *Hsp70* down-regulated and those of *Hsp90* being up-regulated, the injured animals may be undergoing an extended response to unfolded proteins and may have continued stress from protein aggregation.

The Cyclic AMP-responsive element-binding protein may another key regulator of the heat stress response. In the heart of the injured animals, rno-miR-133a-5p and rno-miR-185-5p are up-regulated and are predicted to target Cyclic AMP-responsive element-binding protein 3-like protein 2 (*Creb3l2*), which is down-regulated, while rno-miR-125b-1-3p and rno-miR-152-5p are down-regulated and predicted to target Cyclic AMP-responsive element-binding protein 3-like protein 3 (*Creb3l3*), which is up-regulated. Both *Creb3l2* and *Creb3l3* are thought to be active in times of endoplasmic reticulum stress and activate genes involved in the unfolded protein response [51]. However, *Creb3l3* has also been reported to have a role in maintaining energy homeostasis, which may explain the disparate direction of regulation between the two proteins [52].

#### miR-21

miR-21 has one of the greatest fold change differences in the heart of the injured animals, suggesting it plays an important role in cardiac injury and the heat stress response. miR-21 is one of the most extensively studied miRNA and has previously been implicated in both protective and pathological roles in the heart [53]. We suggest that miR-21 is differentially expressed in response to heat-induced protein damage and disrupted energy metabolism. miR-21 may be furthering an adaptive response in lieu of cell death through interaction with the PI3K-Akt signaling pathway, which has been shown to have cardioprotective effects against ischemia-reperfusion injury [54]. The protective effects occur through the attenuation of apoptosis, which requires Akt phosphorylation. Sayed and colleagues (2010) demonstrated that miR-21 is a regulator of Akt, thereby mediating the anti-apoptotic effects [55]. Indeed, animals with overexpressed miR-21 had smaller infarct size and ameliorated heart failure. Additionally, miR-21 is upregulated or identified as a potential biomarker in many types of cancer and human disease, influencing many cellular pathways processes such as the p53 pathway, cell proliferation, and apoptosis [56]. It has even been suggested to play a role in psychological stress, likely through its role in the regulation of apoptosis [57]. Therefore, miR-21 may be up-regulated in our study diminishing apoptosis through involvement with the Akt signaling pathway, which was enriched in the heart of injured animals.

Furthermore, miR-21 has been shown to have a protective effect against ischaemia/reperfusion injury in rats by reducing cardiac cell apoptosis [58]. Therefore, we tested an in vitro cell model to determine if changing the levels of the mir-21 in H9c2 would enhance or diminish apoptosis in the cells. After 5 h of heat, the cells with decreased levels of miR-21 did show a greater percentage of apoptotic cells than control. This further suggests that miR-21 may play a role in protecting the heart against stress and may serve as an attractive therapeutic.

### Limitations

As with most ‘omics studies, a limitation to this study is the low statistical power due to sample size. This is most evident with our samples at the 48 h time point where the data for the injured and uninjured animals were separated into two groups. While these groupings are consistent with the transcriptomic and metabolic profiles seen in our companion studies [15, 16]. Nevertheless, it was in these injured animals where we found the greatest number of differentially expressed miRNA. However, the reduced statistical power may result in us not identifying these same miRNA as differentially expressed at other time points with larger sample number. The majority of the differentially expressed miRNA we discuss showed limited or no fold change in uninjured animals with larger changes in the injured animals. With limited sample numbers and the use of an FDR designed to limit false positives, we increase the risk of a Type II error and may not have identified all differentially expressed miRNA.

## Conclusions

In our study, we were able to identify differentially expressed miRNA involved in the major functional responses to heat stress in multiple tissues and time points. These miRNA may target mRNAs that are implicated in organ injury, the unfolded protein response, cell signaling, and energy metabolism.

Perhaps one of the most interesting aspects of our study was the ability to distinguish between injured and uninjured animals at 48 h. We were able to identify miRNA and KEGG pathways related to the known effects of heat stress as well as those that indicate organ injury. The differentially expressed miRNA unique to injured animals may indicate the severity of the injury as they gradually increased along the study timeline and had the largest fold change in injured animals. Some of the miRNA that suggest organ injury are known to be released into the plasma and may therefore be candidate biomarkers of organ injury due to heat stress or other causes.

Overall, our study identified miRNA that changed in response to heat stress. We identified pathways that may be regulated by the differentially expressed miRNA using the targeted mRNA data from a companion study. Upon further study, these miRNA may serve as biomarkers for organ injury due to heat stress or as potential therapeutic intervention points or preventatives against heat stress injury.

## Additional files

Additional file 1: Table of histopathology findings. This table describes the histopathology found in the tissues at all time points as published in Stallings 2014. (XLSX 10 kb)

Additional file 2: Differentially expressed miRNA. miRNA were considered differentially expressed with an FDR < 0.05 tested against a fold-change threshold of 1.25 for each condition. This table contains the complete list of differentially expressed miRNA in each condition (XLSX 19 kb)

Additional file 3: mRNA targets. mRNA targets of the modulated miRNA were limited to those changing by at least 1.5 fold in the opposite direction of the perturbed miRNA. (XLSX 158 kb)

Additional file 4: Enriched KEGG Pathways. Complete list of enriched KEGG pathways for each condition. (XLSX 16 kb)
